# A prospective study of extraesophageal reflux and potential microaspiration in patients hospitalized with COVID-19 in Jordan

**DOI:** 10.1186/s12890-023-02638-7

**Published:** 2023-09-12

**Authors:** Hafez Al-Momani, Safaa Mashal, Dua’a Al Balawi, Muna Almasri, Abdel-Ellah AL-Shudifat, Ashraf I Khasawneh, Jeffrey Pearson, Christopher Ward

**Affiliations:** 1 Department of Microbiology, Pathology and Forensic medicine, Faculty of Medicine, The Hashemite University, Zarqa, 13133 Jordan; 2 Faculty of Applied Medical Sciences, The Hashemite University, Zarqa, 13133 Jordan; 3 Department of Internal and family Medicine, Faculty of Medicine, The Hashemite University, Zarqa, 13133 Jordan; 4 Translational and clinical research and Biosciences institutes, Newcastle University Medical School, Newcastle University, Newcastle upon Tyne, NE2 4HH UK

**Keywords:** COVID-19, Gastrointestinal system, COVID-19 transmission, Pepsin, Extraesophageal reflux

## Abstract

**Background:**

Severe acute respiratory syndrome coronavirus 2 (SARS-CoV-2) lung infection has represented a global challenge. Intriguingly, it has been shown that the alveolar lung epithelium expresses little Angiotensin Converting Enzyme receptor protein (ACE2), the entry receptor for SARS-CoV-2. Upper airway establishment of infection and translocation to the lung is well documented but other anatomical niches may be relevant to potentially serious lung infection. ACE2 is heavily expressed in the gastrointestinal tract and gastrointestinal symptoms support a clinical diagnosis of Coronavirus disease 2019 (COVID-19). This suggests a research question and the need to gather patient data exploring potential aerodigestive links in SARS-CoV-2 tranlocation and infection which may be relevant in the peripheral lung. This recognizes anatomical proximity and concepts of bi-directional movement between the Gastrointestinal and lung systems in normal physiology and disease. We have therefore explored the potential for gastro oesophageal reflux disease (GORD) micro aspiration and aeorodigestive pathophysiology in a novel prospective investigation of patients hospitalized with COVID-19.

**Methods:**

This is a prospective descriptive cohort study of 210 patients who were hospitalized with a confirmed diagnosis of COVID-19. The cohort was divided into three groups of patients based on symptom severity and radiological results. The Reflux Symptom Index (RSI) was used to evaluate the presence and severity of GOR. An RSI greater than 13 is considered to be abnormal. Patients’ saliva samples were tested using enzyme-linked immunosorbent assay (ELISA) to determine the level of salivary pepsin among the cohort of patients.

**Results:**

A total of 210 patients with COVID-19 were enrolled in the study with 55.2% (116/210) classified as mildly ill, 31.9% (67/210) moderately ill and 12.9% (27/210) as severely ill. 34% (72/210) of the patients had an RSI score of over 13 and a median salivary pepsin value of 54 ± 29 ng/ml which suggested an incidence of extraesophageal reflux (EOR) in around a third of patients. The presence of respiratory comorbid conditions, an RSI score of over 13 and a salivary pepsin level of > 76ng/ml increased the risk of developing a more severe COVID-19 infection.

**Conclusion:**

The study showed a high prevalence of EOR among the study cohort and provide the first prospective evidence suggesting the potential for aerodigestive pathophysiology including microaspiration in COVID-19 disease. We believe that the results of our study support the need for more extensive research.

**Supplementary Information:**

The online version contains supplementary material available at 10.1186/s12890-023-02638-7.

## Introduction

Coronavirus (CoV) is derived from the Latin word corona, which means “crown” [1]. It causes a variety of respiratory infections in humans, ranging from the common cold to severe respiratory distress syndrome [2, 3]. The current novel CoV disease, also known as severe acute respiratory syndrome (SARS)-CoV-2 and coronavirus disease 2019 (COVID-19), represents an emerging hazard to global health. The COVID-19 epidemic began in the Chinese city of Wuhan around the end of December 2019 swiftly spreading to Thailand, Japan, South Korea, Singapore, and Iran in the first four months [4]. Widespread global viral dissemination occurred and the World Health Organisation declared the COVID-19 a pandemic [5]. After the devastating influenza pandemic of 1918, COVID-19 has unquestionably become one of the world’s most dreaded diseases [4]. COVID-19 has caused tremendous distress among global communities and governments, and its effects have devastated millions of people’s health and economy. It has become a foremost public health concern in the globe.

Initial COVID-19 cases reported in Wuhan, China, are believed to have originated from a zoonotic source at a wholesale seafood market in Huanan that also sold poultry, snakes, bats, and other agricultural animals [6]. For the purpose of isolating the potential virus reservoir, a comprehensive genetic sequence analysis of numerous animal species was conducted [7, 8]. The results indicated that SARS-CoV-2 is a recombinant virus consisting of the bat CoV and a CoV of unknown origin [9]. Based on relative synonymous codon usage (RSCU) on a diversity of animal species, bats were identified as the most likely source of SARS-C0V-2 [10].

From the beginning of the COVID-19 pandemic, it was recognised that combating COVID-19 would require unprecedented collaboration among clinicians, scientists, industries, governments, and other global stakeholders. COVID-19 is primarily a respiratory disease that produces a broad spectrum of symptoms, ranging from asymptomatic patients to critically ill patients with respiratory failure, shock, or multiorgan failure [11] with Ground glass opacity and parenchymal bands/fibrous stripe were the most frequent Chest computed tomography abnormalities [3]. Glucocorticoids have become the mainstay and standard of care for severe COVID-19 according to Mondini, Salton [12]. Glucocorticoids have been shown to reduce mortality and the need for invasive mechanical ventilation in SARS-CoV-2-induced acute respiratory distress syndrome (ARDS) [13]. Vaccines during the covid-19 pandemic were a game-changer in terms of reducing the incidence of SARS-CoV-2 infection, hospitalisation, and mortality due to covid-19 [11].

Prior to this public health measures to limit infections were one of the few options available, and a key goal was to understand and limit the pathophysiology of Severe acute respiratory syndrome coronavirus 2 (SARS-CoV-2) lung infection. There is also growing recognition that COVID-19 is a multi-system disease wherein non-respiratory symptoms and pathophysiology can also occur [14].

It has been shown that COVID-19 can also affect people’s gastro-intestinal (GI) system and exhibit symptoms such as diarrhea, loss of appetite and nausea [15, 16]. Previous studies have found the SARS-CoV-2 RNA in the stool of infected patients. With angiotensin-converting enzyme 2 (ACE2) serving as the viral receptor, SARS-CoV-2 was found to be highly expressed in the GI tract, suggesting that the virus can also infect the digestive system [17, 18].

While lung disease has represented a key global challenge, intriguingly it has been shown that the alveolar lung epithelium expresses little ACE2. Elegant work has shown that very limited alveolar epithelial ACE2 protein expression limits alveolar permissiveness for SARS-CoV-2, and Hönzke, Obermayer [19] concluded that COVID-19 ARDS is “likely caused by secondary immunopathogenesis rather than direct alveolar viral damage”, with SARS-CoV-2 virions, ingested by Alveolar Macrophages strongly implicated [19].

In contrast to the alveolar epithelium ACE2 is heavily expressed in the gastrointestinal tract and gastrointestinal symptoms support a clinical diagnosis of COVID-19. Aspiration is a well documented hazard in the management of Intensive Care Unit patients and (Micro)aspiration by way of gastroesophageal reflux (GOR) is a potential mechanism through which SARS-CoV-2 could spread from the gastro-intestinal tract to the lungs of individuals with ingestion of virions by the professional sentinel phagocytes of the lung. GOR is a mechanism defined by a retrograde flow of stomach contents into the esophagus [20, 21]. GOR can be a physiological process that many people experience normally but it can also be pathological leading to GOR disease (GORD) [22, 23]. Through the application of culture independent methods, previous studies have shown microbiological continuity in the aerodigestive tract of healthy adults indicating that microaspiration of gastric contents may be common in healthy individuals [24].

The term extraesophageal reflux (EOR) refers to gastric contents that spreads beyond the proximal part of the esophagus to the larynx, pharynx or nose [25]. We have previously shown that the human nasal epithelium expresses ACE2 and is especially permissive to SARS-CoV 2 infection and replication [26, 27]. Previous studies showed a link between EOR and various respiratory tract issues manifesting as post-nasal drip, cough, constant throat-clearing, sore throat, tight chest and wheezing [28]. EOR has also been associated with tooth decay and otitis media, with reports of refluxate entering the respiratory passageways [29, 30].

A link between EOR and bacterial colonization of the lower respiratory tract in children with cystic fibrosis (CF) further suggests the potential of gastric immigration and bidirectional transfer of microorganisms [31]. Evidence also suggest that this may be widely relevant in other lung diseases.

A study by Rosen, Hu [32] involving children suffering from chronic cough undergoing bronchoscopy and gastrointestinal endoscopy showed that eight of the most common gastric fluid bacteria were also found flourishing in their lungs. The authors of the study interpreted the result as evidence of a microbiological exchange between the lung and the gastrointestinal tract, independent of the oropharyngeal microbiome [32]. Another study involving asthmatic children with gastroesophageal reflux disease (GERD), marked an increased risk of intestinal and respiratory infection associated with proton-pump inhibitor (PPI) or H2 blocker treatment [33].

In COPD Huang, Liu [34] et al. showed that there was a significant correlation between GERD and COPD exacerbations. We have previously shown that higher pH is associated with the growth of potential respiratory pathogens in the gastric juice of patients [35] and there is an active current debate about COVID-19 risk in people taking PPIs [36].

Several reports offer evidence that a gastric reservoir poses a potential risk for contracting nosocomial pneumonia within the setting of the intensive care unit (ICU) [37]. Our previously published research draws attention to the role of the stomach as a reservoir for some bacterial pathogens [38, 39]. Other studies which involved the elderly, showed a concordance of bacteria between the gut and the respiratory tract. More specifically, a high concentration of the same bacteria in the gut were detected prior to their presence in the respiratory tract [40]. This data supports a link of bacterial colonization between the gastric and the lower respiratory tract [41] locations which could also apply to viral infection and transmission.

GOR disease has been known as one of the most common gastro-intestinal disorders affecting approximately 20% of adults in the western world [42]. A systematic review conducted by El-Serag, Sweet [43] on the prevalence of GERD worldwide showed a rate of 18.1–27.8% in North America, 8.8–25.9% in Europe, 2.5–7.8% in East Asia, 8.7–33.1% in the Middle East, 11.6% in Australia and 23.0% in South America. The true prevalence of this disorder could be higher in reality due to the fact that more and more people have access to over-the-counter acid reducing medications [43, 44].

Past studies showed that the presence of comorbidities in patients diagnosed with COVID-19 resulted in poorer clinical outcomes for these patients [45]. A meta-analysis on the prevalence of comorbidities in patients with COVID-19 revealed hypertension and diabetes as the most common comorbidity, followed by cardiovascular diseases and diseases affecting the respiratory system [45, 46]. However, it is important to note that the comorbidities in these studies were mostly determined through self-reporting upon hospital admission. This could lead to missing data due to the patient’s lack of knowledge and variable awareness of certain conditions. The impact of comorbid digestive system diseases on patients with COVID-19 has not been a common focus of scientific studies and prospective data in particular are lacking.

We have therefore prospectively explored the potential for gastro oesophageal reflux disease (GORD) micro aspiration and aeorodigestive pathophysiology in an investigation of patients hospitalized with COVID-19. We hypothesized that evidence of symptomatic EOR and the detection of the gastric protease pepsin would suggest aerodigestive pathophysiology in patients with COVID-19 and in principle support the potential transmission of SARS-CoV-2 from a gastro intestinal reservoir to the lung.

## Methods

### Study design

This was a cross-sectional cohort study involving 210 patients who were admitted in a single tertiary care hospital in Amman, Jordan. The study was carried out following approval by the hospital’s institutional review board. The patients were hospitalized in Prince Hamza Hospital for isolation and treatment after contracting COVID-19.

### Inclusion and exclusion criteria

Inclusion criteria included an age of ≥ 18 years, and a diagnosis confirmed through laboratory testing for SARS-CoV-2 after undergoing a polymerase chain reaction nasopharyngeal swab test. Patients who were admitted to an intensive care unit (ICU), those who were previously diagnosed with GERD, those who were on acid suppression therapy (proton pump inhibitor, H2 blockers or anti-acid) and those who had a previous history of upper gastro-intestinal disease or surgery were excluded.

The study was conducted over a two-month period from 6 February to 6 April 2022. Patients were asked to sign a consent form upon hospital admission and were interviewed with the help of a trained nurse who collected information from the subjects using a two-part predesigned questionnaire (Table 1).

**Table 1:** The questionnaire used in this study

We would like you to respond to the following questions. The questionnaire is meant to be anonymous. The questionnaire will not be linked to your name.							
1. What is your age?	__________________________(years)						
2. What is your gender?	Male□_________ Female□_						
3. What is your current weight?	__________________________(Kg)						
4. What is your height?	__________________________(cm)						
Are you smoker	Yes□_________ No□_						
Are you alcoholic	Yes□_________ No□_						
**Do you complain any of the following diseases?**							
Coronary artery disease	Yes□_________ No□_						
Congestive heart failure	Yes□_________ No□_						
Cardiac arrhythmia	Yes□_________ No□_						
Hypertension	Yes□_________ No□_						
Hyperlipidemia	Yes□_________ No□_						
Diabetes	Yes□_________ No□_						
Cerebrovascular accident	Yes□_________ No□_						
Pulmonary disorders	Yes□_________ No□_						
Chronic renal insufficiency	Yes□_________ No□_						
Thyroid disorders	Yes□_________ No□_						
Irritable bowel syndrome	Yes□_________ No□_						
Inflammatory bowel disease	Yes□_________ No□_						
Other GI disorders	Yes□_________ No□_						
**Within the last month, how did the following problems affect you? 0 = No problem, 5 = Severe problem**							
		0	1	2	3	4	5
1. Hoarseness or a problem with your voice							
2. Clearing your throat							
3. Excess throat mucus or postnasal drip							
4. Difficulty swallowing food, liquid, or pills							
5. Coughing after you ate or after lying down							
6. Breathing difficulties or choking episodes							
7. Troublesome or annoying cough							
8. Sensation of something sticking in your throat or a lump in your throat							
9. Heartburn, chest pain, indigestion, or stomach acid coming up							
Total (RSI > 13 = Abnormal )							

The first part of the questionnaire features demographic data as well as past medical and surgical history. Patients were asked whether they suffered from any comorbidities such as heart disease, lung disease, gastro-intestinal disease, diabetes mellitus, chronic renal disease and thyroid disorders. They were also asked about tobacco and alcohol consumption. Each patient’s body mass index (BMI) was also calculated using self-reported height and weight information.

The second part of the questionnaire was devoted to the Reflux Symptom Index (RSI). The RSI is used to assess the presence and intensity of commonly reported EOR symptoms [47]. The RSI score for each question ranges from 0 to 5, with 5 being the worst, based on the severity of a number of symptoms. The symptoms listed include: hoarseness or a problem with one’s voice; clearing one’s throat; excess throat mucus or postnasal drip; difficulty swallowing food, liquids, or pills; coughing after eating or after lying down; breathing difficulties or choking episodes; presence of a troublesome or annoying cough; sensations of something sticking in the throat or a lump in the throat; and heartburn, chest pain, indigestion or stomach acid coming up. A score greater than 13 was considered to be clinically significant and indicative of EOR [48]. Both the test-retest reliability (rs = 0.921) and internal consistency reliability (α = 0.969) of the RSI were high [49] .

Other information collected included the patients’ clinical, laboratory and radiological data which were obtained upon hospital admission from patients’ medical reports. Descriptions of chest X-ray or computed tomography (CT) scan reports were also taken from patients’ medical records and evaluated by a radiological consultant who has over ten years clinical experience at Prince Hamza Hospital.

### Patient cohort classification

All the patients enrolled in the study were classified into three groups as being mildly, moderately or severely ill according to the Guidance for Corona Virus Disease 2019 issued by the National Institutes of Health [50]. The three categories of the disease based on severity were defined as follows:

- Mild illness: Individuals who have any of the various signs and symptoms of COVID-19 such as fever, cough, sore throat, malaise, headache, muscle pain, nausea, vomiting, diarrhea, loss of taste and smell but do not have shortness of breath, dyspnea or an abnormal chest result based on chest X-ray or CT scan.
- Moderate illness: Individuals who show evidence of lower respiratory disease during clinical assessment or an abnormal radiological result from a chest X-ray or CT scan and who have an oxygen saturation level (SpO2) of ≥ 94% as measured by pulse oximetry in room temperature at sea level.
- Severe illness: Individuals who show an abnormal radiological result from a chest X-ray or CT scan and display severe symptoms such as a respiratory rate of 30 times per minute or greater, pulse oxygen saturation level of 93% or lower, a rapid progression of pneumonia based on radiological findings within 24 to 48 h.

### Salivary Collection and Pepsin Measurement

Pepsin is one of the main digestive enzymes that aids in the digestion of proteins in food. It is a protease enzyme which is synthesized via its precursor pepsinogen in the stomach’s gastric chief cells. Its presence in the esophagus or more proximal sites is believed to be indicative of reflux [51, 52]. Pepsin was also shown to be present in laryngeal and nasal sinus tissues, tracheal secretions and bronchoalveolar lavage fluid and in saliva. The presence of pepsin in the saliva and/or sputum is a non-invasive diagnostic marker for GERD and considered a biomarker for detecting airway reflux [52–54]. In this study, two milliliters of saliva were collected from each patient into tubes containing 0.5 mL of 0.01 M citric acid. The samples were centrifuged at 4000 rpm for five minutes. Salivary pepsin concentrations were detected using the human pepsin ELISA kit (Catalog No. ELK8433; ELK Biotechnology, Wuhan, China) with detection range at 3.13–200 ng/ml and sensitivity at 0.93 ng/ml. All samples were coded and analyses were carried out blind to all clinical and physiological variables.

### Statistical analysis

The comparison of patients with varying severity of COVID-19 was carried out using univariate analysis, and an *χ*^2^ test or a *t* test was used depending on the type of variable. The researchers used a multivariate logistic regression analysis to identify independent risk factors for COVID-19. P values of < 0.05 were taken to be significant. Data were analyzed using GraphPad InStat 6.0 software.

### Ethical approval

This study was granted ethical approval by the Hashemite University and the Prince Hamza Hospital’s Ethics Service Committee with reference number 5/3/2020/2021. All of the study participants have provided written informed consent prior to their induction into the study. The study was carried out in accordance with the relevant guidelines and regulations.

## Results

### Patient characteristics

A total of 210 patients with COVID-19 were enrolled in this study. Table 2 shows the clinical characteristics of the study participants in detail. The mean age of the patients was 60.7 ± 11.8 years and ranged from 25 to 85 years. 51.4% (108/210) of the patients were male and 48.6% (102/210) were female.

**Table 2:** Demographic and basic clinical characteristics of participants

Characteristic	All patients (n = 210)
**Patient age: mean ± SD (range)**	**60.7 ± 11.8 (25–85)**
**Age group**	
18–28	4 (1.9%)
29–39	8 (3.8%)
40–50	37 (17.9%)
51–61	55 (26.2%)
62–72	61 (29.0%)
73–83	43 (20.5%)
> 83	2 (1.0%)
**Gender**	
Male	108 (51.4%)
Female	102 (48.6%)
**BMI, kg/m**^**2**^, **mean ± SD**	29.0 ± 4.5
**Smokers**	67 (31.9)
**Alcoholic**	17 (8.0%)
**Comorbidity**	122 (58.0%)
Coronary artery disease	39 (18.5%)
Congestive heart failure	24 (11.4%)
Cardiac arrhythmia	8 (3.8%)
Hypertension	57 (27.1%)
Hyperlipidemia	38 (18.1%)
Diabetes	51 (24.3%)
Cerebrovascular accident	11 (5.2%)
Pulmonary disorders	9(4.2%)
Chronic renal insufficiency	19(9.0%)
Thyroid disorders	11(5.2%)
Irritable bowel syndrome	22 (10%)
Inflammatory bowel disease	6 (2.9%)
Other GI disorders	3 (1.4%)

The majority of the patients were classified as overweight to obese with a body mass index (BMI) mean of 29.0 ± 4.5. More than half of the study subjects (143 or 68.1%) were non-smokers and a majority (n = 122, 58.0%) suffered from at least one comorbid condition. The most commonly reported comorbid condition was cardiovascular disease.

### Severity of COVID-19 illness

**Fig. 1:**
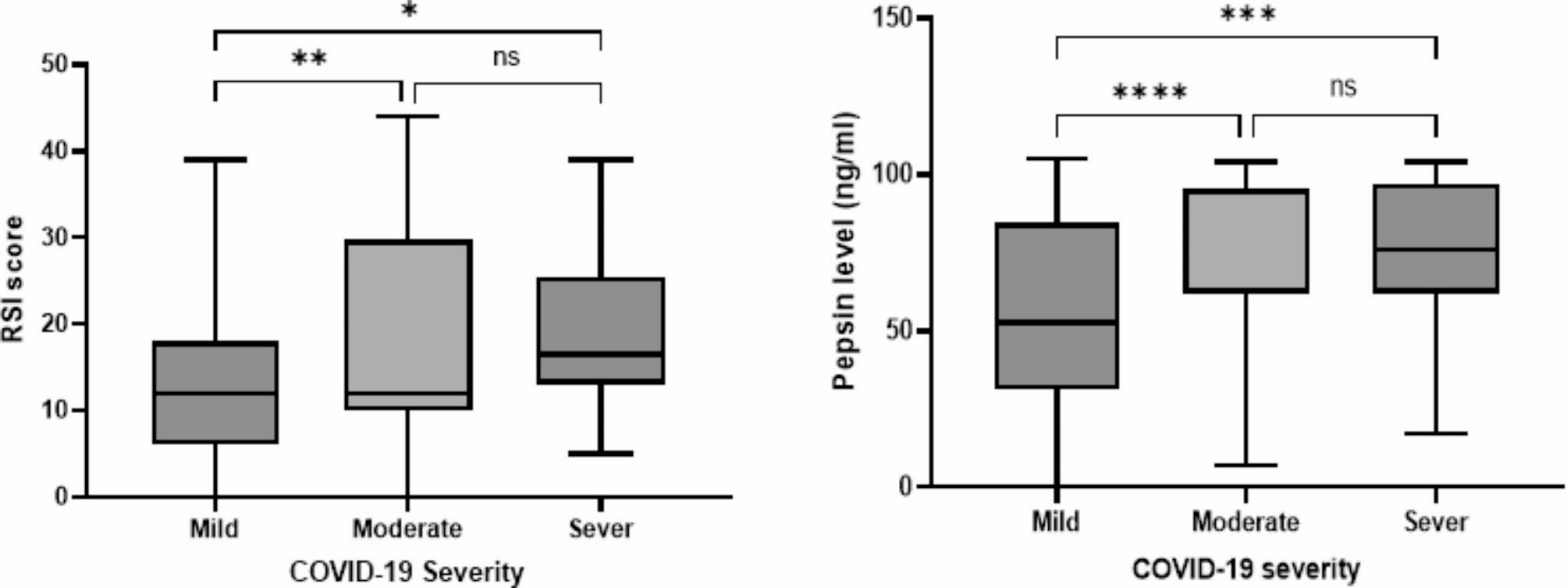
Age and RSI score among different patient’s group. ****<0.0001

The cohort of patients was classified according to the degree of severity of the COVID-19 infection they were suffering from. Based on the criteria for classification of the patient cohort, a total of 116 (55.2%) patients were classified as having a mild COVID-19 illness, 67 (31.9%) with moderate illness and 27 (12.8%) had a severe COVID-19 infection. Table 3 shows the clinical characteristics of these patients. Apart from the BMI, the study found no significant statistical difference among these groups in terms of demography or comorbidity. The study found a statistically significant difference in terms of the BMI between the moderate and severe groups when compared to those in the mild illness group (F (2, 207) = 21.77, P < 0.0001). Please see Fig. 1.

**Table 3:** Demographic and clinical characteristics of participants in the 3 different groups

Characteristic	Mild COVID-1 patient (N = 116, 55.2%)	Moderate COVID-19 patients (N = 67,31.9%)	Severe, COVID-19 patients ill(N = 27,12.8%)	
**Patient age: mean ± SD (range)**	**60 ± 10.6** **(25–80)**	**60.2 ± 13.6** **(35–74)**	**64.9 ± 11.4** **(41–85)**	**P = 0.1473**
**Age group**				
18–28	4 (3.4%)	0 (0.0%)	0 (0.0%)	P = 0.71
29–39	9 (7.8%)	3 (4.5%)	1 (3.7%)	P = 0.50
40–50	15 (12.9%)	14 (20.9%)	4 (14.8%)	P = 0.25
51–61	34 (29.3%)	16 (23.9%)	6 (22.2%)	P = 0.60
62–72	28 (24.1%)	19 (28.4%)	8 (29.6%)	P = 0.50
73–83	25 (21.6%)	15 (22.4%)	7 (25.9%)	P = 0.71
> 83	1 (0.8%)	0 (0.0%)	1 (3.7%)	P = 0.11
**Gender**				
Male	54 (46.6%)	39(58.2%)	15 (55.6%)	p = 0.21
Female	62 (53.4)	28 (41.8%)	12 (44.4%)	
BMI, kg/m^2^, mean ± SD	26.8 ± 4.6	30.2 ± 4.1	32.2 ± 4.5	P < 0.05
Smokers	36 (31.0%)	18(26.8%)	13 (48.1%)	P = 0.12
Alcoholic	9 (7.7%)	6 (8.9%)	2(7.4%)	P = 0.40
**Underlying medical conditions**				
Coronary artery disease	20 (18.1%)	13 (19.4%)	6 (18.5%)	P = 0.49
Congestive heart failure	12 (10.3%)	9 (13.4%)	3 (11.1%)	P = 0.81
Cardiac arrhythmia	5 (4.3%)	2 (3.0%)	1 (3.7%)	P = 0.90
Hypertension	29 (25.0%)	19 (28.4%)	9 (33.3%)	P = 0.65
Hyperlipidemia	18 (15.5%)	15 (22.4%)	5 (18.5%)	P = 0.50
Diabetes	26 (22.4%)	18 (26.9%)	7 (25.9%)	P = 0.77
Cerebrovascular accident	5 (4.3%)	4 (6.0%)	2 (7.4%)	P = 0.76
Pulmonary disorders	2 (1.7%)	3 (4.5%)	4 (14.8%)	P = 0.07
Chronic renal insufficiency	10 (8.6%)	7 (10.4%)	2 (7.4%)	P = 0.8721
Thyroid disorders	5 (4.3%)	5 (7.5%)	1 (3.7%)	P = 0.6073
Irritable bowel syndrome	10 (8.6%)	8 (11.9)	4 (14.8%)	P = 0.64
Inflammatory bowel disease	4 (3.4%)	2 (3.0%)	0 (0.0%)	P = 0.24
Other GI disorders	2(1.7%)	1(1.5%)	0	P = 0.32

**RSI score and salivary pepsin level**.

The RSI was used to assess the presence and intensity of commonly reported EOR symptoms. The mean RSI score for all patients was 13.4 ± 9.9. An RSI score greater than 13 was considered to be abnormal and indicates the presence of EOR. Out of the 210 patients enrolled in the study, 133 (63.3%) had an RSI score below 13 while the rest (n = 77, 36.6%) had an RSI score of more than 13 which was indicative of EOR. Of those who had an RSI score above 13, 34 were assessed as moderately ill, 26 severely ill and 17 were critically ill with COVID-19 (Table 2).

**Fig. 2:**
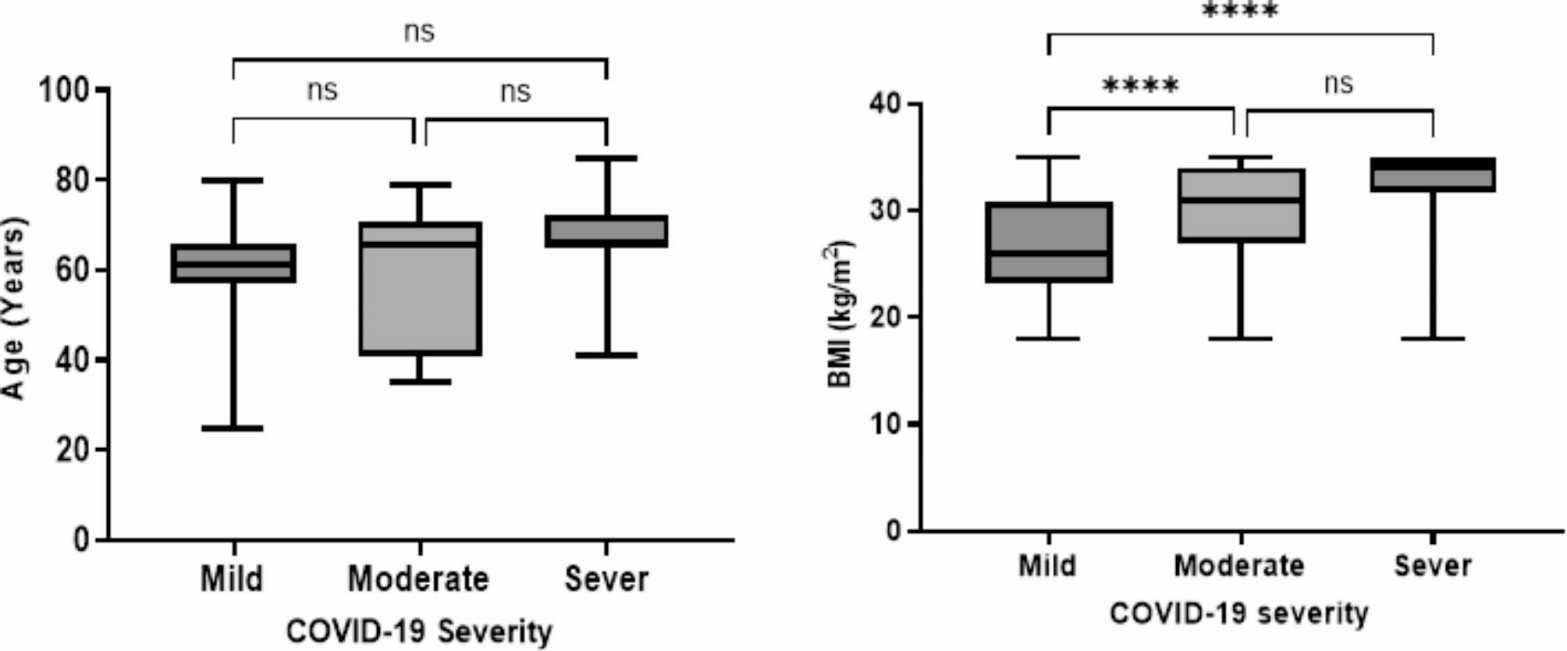
RSI score and level of salivary pepsin among patients included in this study according to COVID-19 severity. ****<0.0001, ***0.0001, **<0.001, *<0.01

The mean RSI score for those patients who were diagnosed with a mild COVID-19 illness was 12.97 ± 8.9, for those with a moderate COVID-19 illness was 18.7 ± 13 and for those who were severely ill was 19.2 ± 9.4. They also showed a significant difference of (*P* = 0 0.005). Figure 2 demonstrates the different RSI scores among the three groups. The RSI scores of both the moderate and severe COVID-19 patient groups were significantly higher than those of the mildly ill patients (F (2, 206) = 7.835, P = 0.0005).

With regards to the patients’ salivary pepsin level as detected by ELISA, the mean pepsin level for all patients was 69.4 ± 26.9ng/ml with a range of 0-104ng/ml and a median of 76ng/ml. A total of five patients had undetectable levels (Table 4). The study found some statistically significant differences on the level of pepsin detected among the different COVID-19 patient groups (F (2, 206) = 23.84, P < 0.0001). Both the moderate and severe COVID-19 patient groups showed a higher level of salivary pepsin compared to the mildly ill group (Fig. 2). Overall, these results suggest that a higher level of salivary pepsin as well as a high RSI score of over 13 may be associated with severe or critical expression of COVID-19 disease.

**Table 4:** RSI score ad Pepsin level among patient included in this study cohort,

	All patient	Mildly ill (N = 116, 55.2%)	moderately ill(N = 67, 31.9%)	severely ill(N = 27,12.9%)	P value
**RSI Score**					
0–13	133 (63.3%)	82 (70.7%)	41 (61.2%)	10 (37.0%)	P = 0.004
> 13	77 (36.6%)	34 (29.3%)	26 (38.8%)	17 (62.9%)	
Mean + SD	13.4 ± 9.9	12.97 ± 8.9	18.72 ± 13	19.23 ± 9.4	P = 0.0005
range	0–44	0–39	0–44	5–39	
**Pepsin level**					
Mean + SD	69.4 ± 26.9	54.4± 27.5	81.2± 22.0	75.8± 25.3	P < 0.0001)
Range	25–104	0-105	7-104	17–104	
Below median 76ng/ml (N)%	108 (51.4%)	79 (68.0%)	18 (26.8%)	10 (37.0%)	< 0.0001
Above median 76ng/ml, (N)%	102 (48.5%)	37 (31.8%)	49 (73.0%)	17 (62.9%)	

### The correlation of RSI scores with the severity of COVID-19

**Fig. 3:**
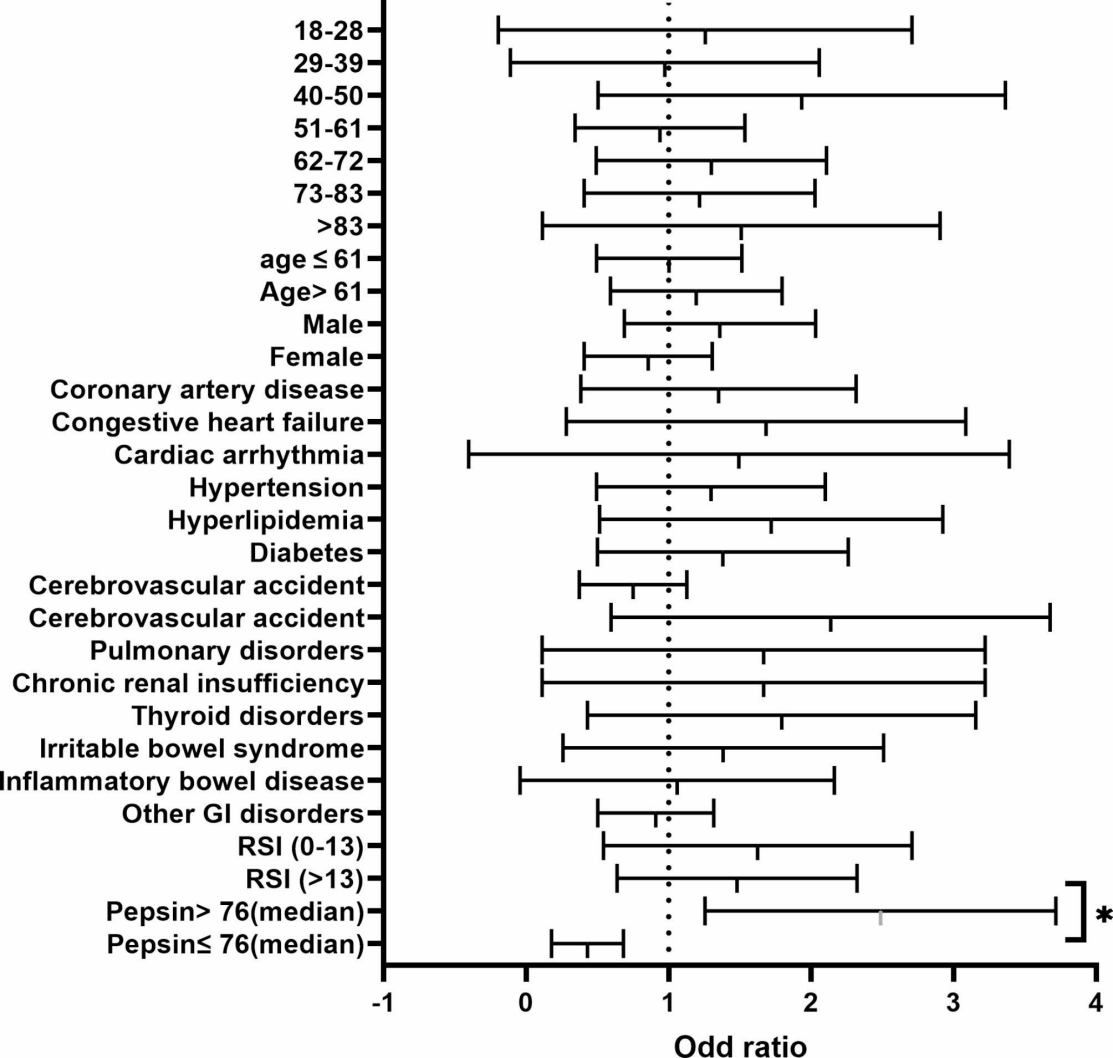
Risk factor for development moderate COVID-19 infection. ****<0.0001, ***0.0001, **<0.001, *<0.01

The study showed that factors such as the patient’s age, gender, BMI, comorbidity and RSI score were not linked to an increased risk of developing a moderate level of COVID-19 illness. However, a patient’s salivary pepsin level was significantly associated with an increased risk of developing a moderate level of COVID-19 infection (Odd ratio (OR) 2.3; 95% CI, 1.36–3.8) (Fig. 3, supplementary Table 1).

**Fig. 4:**
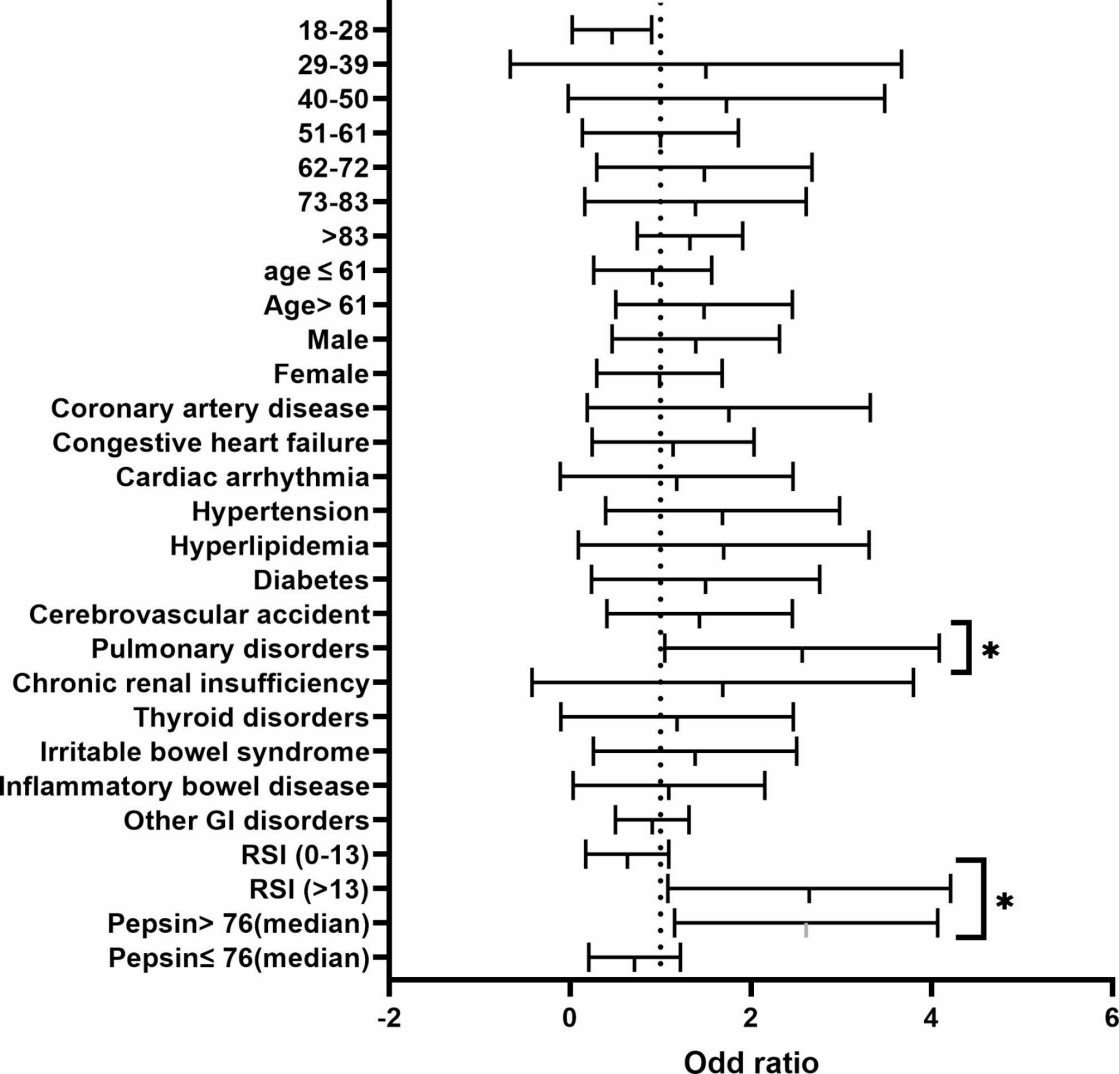
Risk Factors for development severe COVID − 19 infection. *<0.01

Factors such as the presence of respiratory comorbidity, an RSI score of more than 13 and a salivary pepsin level of more than 76ng/ml were associated with an increased risk of developing a severe COVID-19 infection. In a multivariable model, patients who had a respiratory system comorbidity had a statistically significant increased risk of developing a more severe COVID-19 infection (*P* < 0.05; OR = 2.3; 95% CI, 1.04–4.9). Patients who had an RSI score of more than 13 are 2.14 times more likely to experience a severe COVID-19 illness than those with an RSI score of less than 13 (*P* < 0.01; OR = 2.14; 95% CI, 1.04–4.4). In addition, patients who have a salivary pepsin level of more than 76ng/ml (median) are two times more likely to contract a severe COVID-19 illness than those with a salivary pepsin level of less than 76ng/ml (OR = 2.08; 95% CI, 1.07–4.26). (Fig. 4, supplementary Table 2.)

## Discussion

In Jan 2023, there were approximately 667 million confirmed cases of COVID-19 in the world with 6.7 million people reported to have lost their lives to the pandemic. Sadly, this figure is liable to be an underestimate of the true burden and the number of total cases and deaths attributable to COVID-19 continues to rise. Although most people only experience a few symptoms or a mild to moderate illness, a large minority of the population remain at a higher risk of contracting more severe disease. This may require hospitalization and a significant number of patients suffer from morbidity, adverse outcomes such as mechanical ventilation and even death, often as a result of respiratory ARDS [55].

The aim of this study was to explore the potential for reflux associated micro aspiration and aeorodigestive pathophysiology in a novel prospective investigation of patients hospitalized with COVID-19. It is well established that SARS-CoV-2 infection in the gastrointestinal system can occur. Our study has therefore explored in principle the potential for translocation of SARS-CoV-2 from a gastric reservoir to the lung. As far as we know, our study is the first investigation on the prevalence of EOR in patients hospitalized with COVID-19 and its association with the severity of COVID-19 infection. The study used the RSI, a nine-item symptom instrument, to assess the presence of EOR (i.e. symptoms associated with retrograde refluxate leaving the oesophagus), which tends to be more accurate and valid than self-recall [48]. This was complemented by the use of salivary pepsin level as a marker for EOR [56].

The study has shown that among patients who were hospitalized with COVID-19, high levels of salivary pepsin and an RSI score of greater than 13 which, which is indicative of EOR, correlated with poorer clinical outcomes. It also showed that the prevalence of EOR among COVID-19 patients may be higher than in the general population. A potential mechanism for increased EOR could be that COVID-19 may impair both upper and lower esophageal sphincter (ES) function and could aggravate reflux. At a practical level the evaluation of patient EOR status by determining their RSI and salivary pepsin levels could help healthcare professionals with risk stratification of patients upon hospital admission and was shown to be practicable in a real world hospital setting in this study.

Given the worldwide importance COVID-19 there has been a massive global research effort aimed at mapping the expression of ACE2 in patient derived samples as this is widely recognized receptor for SARS-CoV-2. Many studies have evaluated the expression of mRNA with fewer looking at protein based evidence of receptor expression. Intriguingly the current literature is consistent with the lung alveolar epithelium having very little expression of ACE2. It has been concluded that low ACE2 expression means that alveolar permissiveness for SARS-CoV-2 is limited and that uptake of virus by lung phagocytes and in particular alveolar macrophages may lead to immune activation and severe disease. It is currently postulated that COVID-19 ARDS may therefore be attributable to secondary immunopathogenesis [19].

Our study found that approximately 34.3% (72/210) of the patients hospitalized with COVID-19 had an RSI score greater than 13 and a mean salivary pepsin level of 76ng/ml which suggested that a large number of patients may have had EOR as a comorbid condition. The prevalence of EOR in Asia and the Middle East was estimated to be between 10 and 30% [57]. Viewed against this context, our study result implies that the prevalence of EOR in COVID-19 patients may be higher than in the general population, as those who were previously diagnosed with GERD, those who were on acid suppression therapy (proton pump inhibitor, H2 blockers or anti-acid) and those who had a previous history of upper gastro-intestinal disease or surgery were prospectively excluded from our study.

The high prevalence of EOR among the COVID-19 patients suggests that SARS-CoV-2 may affect the motility of the GI tract in patients with COVID-19 or that it may have been caused by the potential impact of the virus on esophageal sphincter tone. However, this hypothesis remains to be studied and further work is needed to explore the impact of SARS-CoV-2 on esophageal physiology.

Currently, there are two lines of thinking which may explain the increase of the severity of COVID-19 infection in patients with EOR namely, the Reflex Theory and Reflux Theory [58]. According to the Reflex Theory, the vagus nerve is stimulated by the gastric reflux in the oesophagus which leads to bronchospasms. This then increases bronchial hyper-responsiveness which in turn leads to adverse effects on the airways [59]. The Reflux Theory, on the other hand, suggests that gastric refluxate is aspirated which causes the airways to become damaged and inflamed exacerbating the damaging effects caused by COVID-19 [60]. In an early pandemic study it was shown that 6 of 13 critically ill patients had gastric fluid that was PCR positive for SARS-CoV-2 [61] and SARS-CoV-2 can remain viable in simulated gastric and intestinal fluids between pH 3–7 [62]. The reflux of duodenal contents into the stomach is a physiological process which mainly occurs after a meal [63]. It has also been shown using radiotracer methodology that micro aspiration of non-sterile gastric contents may occur in normal individuals [64, 65].

A recent report by the US Centers for Disease Control and Prevention (CDC) documented evidence of an infectious virus, not just the viral RNA, in the stool of a patient with severe COVID-19 disease [66]. Similarly, another study also described finding “live” virus in the feces [67]. Other reports stated that nearly half of patients with COVID-19 have viral RNA in their stools even at times when these are not concurrently found in their respiratory tract [68]. Taken together, this body of research in addition to other studies [69, 70], strongly implicates the GI system as a major portal and reservoir for viable SARS-CoV-2 infection with the added possibility of fecal-oral or gut to lung transmission of COVID-19 [16]. In principle our study is potentially consistent with SARS CoV-2 being aspirated into the lungs from a gastric reservoir and this should be investigated further.

Our previous research has suggested a correlation between gastric juice microflora and sputum samples from Cystic fibrosis (CF) cases, suggesting a possible significant gastric infection etiology in CF and a potential reservoir of microbes [71, 72]. In a study of CF cases among children, Palm, Sawicki [31] et al. showed a correlation between lower-airway *pseudomonas aeruginosa* infection and GOR, thereby further reinforcing the theory of microbial transfer between the respiratory and gastro-intestinal systems. A more recent study also suggested a microbial transfer between the GI tract and the lungs independent of the oropharyngeal tract flora [32]. This particular study which involved pediatric cases of chronic cough that were subjected to gastro-intestinal endoscopy and bronchoscopy showed that eight of the most common microbes in gastric fluid samples were also present in the lungs.

Almario, Chey [36] et al. showed that the previous use of proton-pump inhibitors (PPIs) is linked to an increased risk of death from COVID-19. This association was supported by a meta-analysis of eight studies [73] which demonstrated that previous use of PPIs increases the risk of progression to a severe expression of COVID-19. Almario, Chey [36] et al. also found that individuals who used PPIs had higher odds of testing positive for COVID-19 when compared to those who were non-PPI users. Their hypothesis was that PPI use could increase the risk of contracting COVID-19 by undermining the gastric barrier. This is in addition to the increased ability of the virus to survive in an environment of low gastric acidity, creating a niche that could be refluxed, with microaspiration into the respiratory system. More extensive studies are clearly needed to determine the impact of PPIs on the survival of SARS-CoV-2 in the gastric environment. This is particularly important as hypochlorhydria or low stomach acid is quite common among the elderly [74], a segment of the population who are at a significantly higher risk of contracting COVID-19.

Xiao, Chakraborti [75] et al. found that under neutral pH circumstances, the S protein of SARS-CoV aided in the virus’ fusion with host cells. SARS-CoV can stay stable within a range of neutral pH values, however Darnell, Subbarao [76] et al. verified in their study that strongly alkaline, pH 12 and 14, and highly acidic, pH 1 and 3, conditions may lead to the deactivation of the virus. At pH values between 5 and 9, SARS-CoV-2 was still alive on day 6, but had lost between 2.9 and 5.33 logs of infectivity. SARS-CoV-2 was rendered noninfectious in less than a day at pH 2–3 and pH 11–12 [77, 75]. Zhou, Niu [78] et al. used viruses pseudotyped with SARS-CoV-2 S protein to demonstrate that the virus was entirely inactivated and unable to infect cells in very acidic conditions of pH 1.0 and 2.0, equivalent to the usual acidity of an empty stomach.

The acidity of stomach gastric juices ranges from 1 to 3, whereas that of the small and large intestines is between 7 and 8. Most viruses, including SARS-CoV-2, are killed by stomach acid in a natural environment. However, long-term use of an acid suppressant such as a proton pump inhibitor may result in a less acidic stomach environment. In this instance, the SARS-CoV-2 virus has a better opportunity to reach the intestines through the stomach, increasing the likelihood of viral infection and the subsequent risk of developing gastrointestinal symptoms.

We believe that this study is the first prospective attempt to evaluate EOR and the presence of a biomarker of micro aspiration in patients with COVID-19 and provides new information. However, our study is exploratory and has limitations. This was a single-center observational study of hospitalized patients which cannot accurately reflect all patients diagnosed with COVID-19. In addition, the sample size of the study was relatively small. We recommend that subsequent studies should involve a larger population size to increase the generalizability of the data. One other limitation was that the study only included patients who were hospitalized with COVID-19 and thus were likely to present a more severe expression of the disease than patients who were not hospitalized thereby resulting in selection bias. The authors of this study are also aware that the use of RSI > 13 as a diagnostic tool has been debated. The RSI includes non-specific symptoms such as hoarseness, cough, throat clearing, sticky mucus, etc. which are also common in many inflammatory diseases of the upper aerodigestive tract including allergy, rhinitis or chronic rhinosinusitis and pharyngolaryngitis [79–81]. The RSI was not designed to confirm an EOR diagnosis in isolation but to predict an EOR diagnosis when used in combination with other supportive findings [81]. This was one reason why the authors included the use of a non-invasive objective approach in the present study by way of measuring the salivary pepsin level in order to increase the accuracy of the EOR detection. Given the acute challenge represented by performing research studies in a pandemic, where aerosol generation was a known hazard, it was necessary to make a practical compromise regarding the investigation of EOR, with these suitable for the patient population, some of whom were very unwell. Moreover, it may also be deemed inappropriate to utilise more specialised investigative techniques, such as flexible endoscopy or ambulatory 24-hour double-probe pH monitoring, since they are both very expensive and invasive.

Despite its limitations, this study considering potential GOR micro aspiration and aeorodigestive pathophysiology in patients hospitalized with COVID-19 is novel. Our understanding as to which conditions increase the risk of a COVID-19 serious illness and their contribution to adverse outcomes is still developing. A number of studies have shown that patients with comorbidities are more likely to have a severe infection and poorer clinical outcomes [82–84]. If SARS CoV-2 microaspiration challenge of the lung is involved in COVID-19 pathophysiology this may represent a treatable trait, and a risk that may be modifiable by a range of safe existing strategies. If confirmed, the results of this study could aid in providing a more comprehensive assessment on the prognosis of patients hospitalized with COVID-19. The results of the study suggest a possible association of EOR with a more severe disease. More specifically, our study has demonstrated that a patient’s RSI score and level of salivary pepsin together with the presence of respiratory comorbidity are risk factors to developing a more severe COVID-19 disease. Our study results therefore add to the current body of evidence showing the impact of EOR, a common GI disorder, on patients diagnosed with COVID-19.

### Electronic supplementary material

Below is the link to the electronic supplementary material.

Supplementary Material 1

## Data Availability

All data generated or analysed during this study are included in this published article and its supplementary information files.
