# Supplementary material for: A prospective study of extraesophageal reflux and potential microaspiration in patients hospitalized with COVID-19 in Jordan
Source: BMC Pulm Med. 2023 Sep 12;23:341. doi: 10.1186/s12890-023-02638-7 (PMC10496175; doi:10.1186/s12890-023-02638-7)
Supplement: Supplementary file 1 — Supplementary Material 1 [file 12890_2023_2638_MOESM1_ESM.docx]

**A Prospective Study of Extraesophageal Reflux and Potential Microaspiration in Patients Hospitalized with COVID-19 in Jordan:**

Hafez Al-Momani^a^, Safaa Mashal^a^, Dua’a Al Balawi^b^, Muna Almasri^b^, Abdel-Ellah AL-Shudifat^c^, Ashraf I Khasawneh^a^, Jeffrey Pearson^d^, Christopher Ward^d^.

| **Characteristic** | **Mild illness group (N=116, 55.2%)** | **Moderate illness group (N=67,31.9%)** | **Odd ratio** | **P value** |
| --- | --- | --- | --- | --- |
| **Age group** |  |  |  |  |
| 18-28 | 4 (3.4%) | 0 (0.0%) | 0.7259 (0.03-7.9) | P = 0.38 |
| 29-39 | 9 (7.8%) | 3 (4.5%) | 0.57 (0.15 - 2.20) | P = 0.42 |
| 40-50 | 15 (12.9%) | 14 (20.9%) | 1.6 (0.7-3.5) | P = 0.23 |
| 51-61 | 34 (29.3%) | 16 (23.9%) | 0.81 (0.42 - 1.59) | P = 0.55 |
| 62-72 | 29 (25.0%) | 19 (28.4%) | 1.13 (0.59 - 2.18) | P = 0.71 |
| 73-83 | 25 (21.6%) | 15 (22.4%) | 1.04 (0.51 - 2.10) | P = 0.92 |
| >83 | 1 (0.8%) | 0 (0.0%) | 1.7259 (0.03-2.8) | P = 0.78 |
| age ≤ 61 | 62 (53.4%) | 33 (49.2%) | 0.9215 (0.54-1.55) | P = 0.75 |
| Age> 61 | 54 (46.5%) | 34 (50.7) | 1.09 (0.65-1.84) | P = 0.74 |
| **Gender** |  |  |  |  |
| Male | 54 (46.6%) | 39 (58.2%) | 1.25 (0.75 - 2.08) | P = 0.39 |
| Female | 62 (53.4) | 28 (41.8%) | 0.78 (0.45 - 1.34) | 0.37 |
| **Underlying medical conditions** |  |  |  |  |
| Coronary artery disease | 20 (18.1%) | 13 (19.4%) | 1.12 (0.52 - 2.41) | P = 0.76 |
| Congestive heart failure | 12 (10.3%) | 9 (13.4%) | 1.29 (0.52 - 3.24 | P = 0.57 |
| Cardiac arrhythmia | 5 (4.3%) | 2 (3.0%) | 0.69 (0.13 to 3.66) | P = 0.66 |
| Hypertension | 29 (25.0%) | 19 (28.4%) | 1.13 (0.59 - 2.17) | P = 0.70 |
| Hyperlipidemia | 18 (15.5%) | 15 (22.4%) | 1.44 (0.68 - 3.04) | P = 0.34 |
| Diabetes | 26 (22.4%) | 18 (26.9%) | 1.19 (0.61 - 2.34) | P = 0.59 |
| Cerebrovascular accident | 5 (4.3%) | 4 (6.0%) | 1.38 (0.35 - 5.33) | P = 0.63 |
| Pulmonary disorders | 2 (1.7%) | 3 (4.5%) | 1.2 (0.4-4.9) | P = 0.3 |
| Chronic renal insufficiency | 10 (8.6%) | 7 (10.4%) | 1.2 (0.4-3.4) | P = 0.70 |
| Thyroid disorders | 5 (4.3%) | 5 (7.5%) | 1.7 (0.48-6.2) | P = 0.34 |
| Irritable bowel syndrome | 10 (8.6%) | 8 (11.9%) | 1.4 (0.25-7.5) | P = 0.66 |
| Inflammatory bowel disease | 4 (3.4%) | 2 (3.0%) | 0.86 (0.15-4.8) | P = 0.87 |
| Other GI disorders | 2 (1.7%) | 1 (1.5%) | 0.86 (0.07-9.7) | P = 0.90 |
| **RSI score** |  |  |  |  |
| 0-13 | 82 (70.7%) | 41 (61.2%) | 0.86 (0.53 - 1.34) | P = 0.55 |
| >13 | 34 (29.3%) | 26 (38.8%) | 1.32 (0.73 - 2.39 | P = 0.35 |
| Pepsin level |  |  |  |  |
| Below median 76ng/ml | 79 (68.0%) | 18 (26.8%) | 0.39 (0.2-0.7 | P = 0.0021 |
| Above median 76ng/ml | 37 (31.8%) | 49 (73.0%) | 2.3 (1.36-3.8 | P = 0.0018 |

**Supplementary table 1: Risk factor for development moderate COVID-19 infection**

| **Characteristic** | **Mild illness group (N=116, 55.2%)** | **Sever illness group (N=27,12.8%)** | **OR** | **P value** |
| --- | --- | --- | --- | --- |
| **Age group** |  |  |  |  |
| 18-28 | 4 (3.4%) | 0 (0.0%) | 0.47 (0.02-9.0) | P = 0.61 |
| 29-39 | 9 (7.8%) | 1 (3.7%) | 0.47(0.05 - 3.92) | P = 0.49 |
| 40-50 | 15 (12.9%) | 4 (14.8%) | 1.14 (0.35-3.7) | P = 0.82 |
| 51-61 | 34 (29.3%) | 6 (22.2%) | 0.75(0.29 - 1.98) | P = 0.57 |
| 62-72 | 29 (25.0%) | 8 (29.6%) | 1.18(0.48 to 2.87) | P = 0.70 |
| 73-83 | 25 (21.6%) | 7 (25.9%) | 1.03 (0.38 to 2.75) | P = 0.95 |
| >83 | 1 (0.8%) | 1 (3.7%) | 1.24 (0.35-2.7) | P=0.3 |
| age ≤ 61 | 62 (53.4%) | 11 (40.7%) | 0.76 (0.35-1.63) | P = 0.48 |
| Age> 61 | 54 (46.5%) | 16 (59.2) | 1.27 (0.63-2.55) | P = 0.49 |
| **Gender** |  |  |  |  |
| Male | 54 (46.6%) | 15 (55.6%) | 1.19 (0.58-2.4) | P = 0.62 |
| Female | 62 (53.4) | 12 (44.4%) | 0.83 (0.39 - 1.75 | P = 0.62 |
| **Underlying medical conditions** |  |  |  |  |
| Coronary artery disease | 20 (18.1%) | 6(18.5%) | 1.3 (0.47-3.5) | P =0.62 |
| Congestive heart failure | 12(10.3%) | 3 (11.1%) | 1.07 (0.28-4.07) | P =0.91 |
| Cardiac arrhythmia | 5 (4.3%) | 1 (3.7%) | 0.85 (0.096-7.6) | P =0.89 |
| Hypertension | 29 (25.0%) | 9 (33.3%) | 1.4 (0.56-3.1) | P =0.51 |
| Hyperlipidemia | 18 (15.5%) | 5 (18.5%) | 1.2 (0.4-3.5) | P =0.75 |
| Diabetes | 26 (22.4%) | 7 (25.9%) | 1.15 (0.45-2.9) | P =0.76 |
| Cerebrovascular accident | 5 (4.3%) | 2 (7.4%) | 1.7 (0.3-9.3) | P =0.53 |
| Pulmonary disorders | 2 (1.7%) | 4 (14.8%) | 2.3(1.2- 4.20) | P < 0.01 |
| Chronic renal insufficiency | 10 (8.6%) | 2 (7.4%) | 0.8 (0.17-4.1) | P =0.85 |
| Thyroid disorders | 5 (4.3%) | 1 (3.7%) | 0.86 (0.09-7.6) | P=0.89 |
| Irritable bowel syndrome | 10 (8.6%) | 4 (14.8%) | 1.4 (0.25-2.5) | P =0.67 |
| Inflammatory bowel disease | 4 (3.4%) | 0 (0.0%) | 0.86 (0.17-2.25) | P =0.85 |
| Other GI disorders | 2 (1.7%) | 0 | 0.86 (0.53-1.34) |  |
| RSI score |  |  |  |  |
| 0-13 | 82 (70.7%) | 10 (37.0%) | 0.52 (0.24-1.14) | P=0.10 |
| >13 | 34 (29.3%) | 17 (62.9%) | 2.14 (1.04 - 4.40) | P = 0.03 |
| Salivary pepsin level |  |  |  |  |
| Below median 76 | 79 (68.0%) | 10 (40.7%) | 0.59 (0.28-1.27) | P = 0.18 |
| Above median 76 | 37 (31.8%) | 17 (59.2%) | 2.08 (01.02-4.26) | P = 0.04 |

**Supplementary table 2. Risk Factors for development severe COVID -19 infection**
